# Supplementary material for: Comparative analysis of the Mexico City Prospective Study and the UK Biobank identifies ancestry-specific effects on clonal hematopoiesis
Source: Nat Genet. 2025 Feb 13;57(3):572–82. doi: 10.1038/s41588-025-02085-6 (PMC11906367; doi:10.1038/s41588-025-02085-6)
Supplement: Supplementary file 1 — Supplementary Figs. 1–8, Supplementary Methods, Supplementary Notes 1–3, Supplementary References. [file 41588_2025_2085_MOESM1_ESM.pdf]

nature genetics

Supplementary information https://doi.org/10.1038/s41588-025-02085-6

# Comparative analysis of the Mexico City Prospective Study and the UK Biobank identifies ancestry-specific effects on clonal hematopoiesis

In the format provided by the authors and unedited

## Table of Contents

Supplemental Figures ........................................................................................ 2
Supplementary Methods .................................................................................... 19
Supplemental Notes ........................................................................................... 21
Supplementary References ................................................................................ 25

## Supplemental Figures

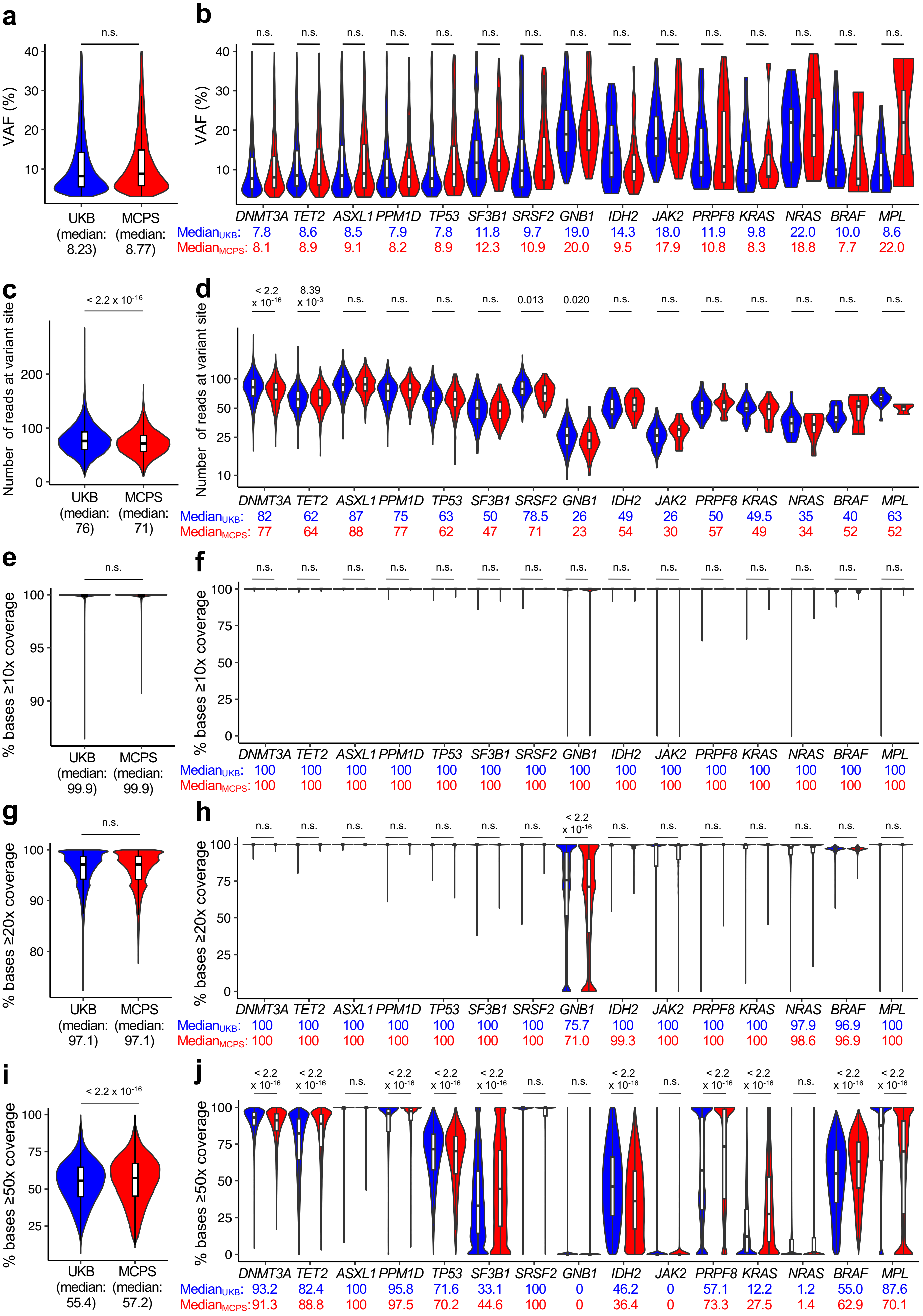
a
n.s.
VAF (%)
UKB (median: 8.23)
MCPS (median: 8.77)
b
VAF (%)
DNMT3A TET2 ASXL1 PPM1D TP53 SF3B1 SRSF2 GNB1 IDH2 JAK2 PRPF8 KRAS NRAS BRAF MPL
Median$_{UKB}$: 7.8 8.6 8.5 7.9 7.8 11.8 9.7 19.0 14.3 18.0 11.9 9.8 22.0 10.0 8.6
Median$_{MCPS}$: 8.1 8.9 9.1 8.2 8.9 12.3 10.9 20.0 9.5 17.9 10.8 8.3 18.8 7.7 22.0
c
< 2.2 x $10^{-16}$
Number of reads at variant site
UKB (median: 76)
MCPS (median: 71)
d
< 2.2 x $10^{-16}$ 8.39 x $10^{-3}$ n.s. n.s. n.s. n.s. 0.013 0.020 n.s. n.s. n.s. n.s. n.s. n.s. n.s.
Number of reads at variant site
DNMT3A TET2 ASXL1 PPM1D TP53 SF3B1 SRSF2 GNB1 IDH2 JAK2 PRPF8 KRAS NRAS BRAF MPL
Median$_{UKB}$: 82 62 87 75 63 50 78.5 26 49 26 50 49.5 35 40 63
Median$_{MCPS}$: 77 64 88 77 62 47 71 23 54 30 57 49 34 52 52
e
n.s.
% bases ≥10x coverage
UKB (median: 99.9)
MCPS (median: 99.9)
f
% bases ≥10x coverage
DNMT3A TET2 ASXL1 PPM1D TP53 SF3B1 SRSF2 GNB1 IDH2 JAK2 PRPF8 KRAS NRAS BRAF MPL
Median$_{UKB}$: 100 100 100 100 100 100 100 100 100 100 100 100 100 100 100
Median$_{MCPS}$: 100 100 100 100 100 100 100 100 100 100 100 100 100 100 100
g
n.s.
% bases ≥20x coverage
UKB (median: 97.1)
MCPS (median: 97.1)
h
n.s. n.s. n.s. n.s. n.s. n.s. n.s. < 2.2 x $10^{-16}$ n.s. n.s. n.s. n.s. n.s. n.s. n.s.
% bases ≥20x coverage
DNMT3A TET2 ASXL1 PPM1D TP53 SF3B1 SRSF2 GNB1 IDH2 JAK2 PRPF8 KRAS NRAS BRAF MPL
Median$_{UKB}$: 100 100 100 100 100 100 100 75.7 100 100 100 100 97.9 96.9 100
Median$_{MCPS}$: 100 100 100 100 100 100 100 71.0 99.3 100 100 100 98.6 96.9 100
i
< 2.2 x $10^{-16}$
% bases ≥50x coverage
UKB (median: 55.4)
MCPS (median: 57.2)
j
< 2.2 x $10^{-16}$ < 2.2 x $10^{-16}$ n.s. < 2.2 x $10^{-16}$ < 2.2 x $10^{-16}$ < 2.2 x $10^{-16}$ n.s. n.s. < 2.2 x $10^{-16}$ n.s. < 2.2 x $10^{-16}$ < 2.2 x $10^{-16}$ n.s. < 2.2 x $10^{-16}$ < 2.2 x $10^{-16}$
% bases ≥50x coverage
DNMT3A TET2 ASXL1 PPM1D TP53 SF3B1 SRSF2 GNB1 IDH2 JAK2 PRPF8 KRAS NRAS BRAF MPL
Median$_{UKB}$: 93.2 82.4 100 95.8 71.6 33.1 100 0 46.2 0 57.1 12.2 1.2 55.0 87.6
Median$_{MCPS}$: 91.3 88.8 100 97.5 70.2 44.6 100 0 36.4 0 73.3 27.5 1.4 62.9 70.1

**Supplementary Fig. 1 | Variant allele frequency and sequencing coverage comparison of clonal haematopoiesis (CH) genes between UK Biobank (UKB) and Mexico City Prospective Study (MCPS). a,b**, Comparison of variant allele frequency (VAF) between UKB and MCPS for all CH driver gene variants (**a**) and for gene-specific CH driver gene variants **(b)**. *P* values were derived from linear regression with age as a co-variate. **c,d**, Number of sequencing reads at site of variants for all CH driver gene variants (**c**) and for gene-specific CH driver gene variants (**d**). Data points in **(a,c)** represent the VAF for all 15 CH genes across all samples with CH driver gene mutations. In total, 4,249 MCPS and 20,488 UKB participants with CH driver gene mutations were included for analysis here **(a-d)**. **e-j**, Percentage of bases with at least 10x (**e-f**), 20x (**g-h**), and 50x coverage (**i,j**) across all targeted regions of CH driver genes (**e,g,i**) and for gene-specific CH driver genes (**f,h,j**). Data points in **(e,g,i)** represent the average percentage of bases covered for all 15 CH gene across all samples included in this study. Two-sided *P* values for coverage at variant sites and percentage of bases covered were derived from Wilcoxon ranked sum test. *P* values were adjusted for multiple testing using Benjamini-Hochberg procedure. Boxplots represent the median, first and third quartiles, and whiskers represent 1.5 times the interquartile range **(c-j)**. In total, 136,401 MCPS and 416,118 UKB participants irrespective of CH driver gene mutations detected were included for analysis here **(e-j).** For the following genes, only specific regions of the isoforms were assessed for coverage: *ASXL1* (exons 12 and 13), *PPM1D* (exons 5 and 6), *SF3B1* (exons 14 and 15 in which the K666 and K700 hotspot variants are located, respectively), *SRSF2* (exon 1 in which the P95 hotspot variant is located), *GNB1* (exon 5 in which the K57 hotspot variant is located), *JAK2* (exon 14 in which the V617F hotspot variant is located), *PRPF8* (exon 31 in which the C1594 and D1598 hotspot variants are located), and *MPL* (exon 10 in which the W515 hotspot variant is located). For all other genes, the entire coding region of the isoform was assessed for coverage. *P* value ** < 0.01 * < 0.05 for overall CH. FDR ** < 0.01 * < 0.05 for gene-specific CH. CH, clonal haematopoiesis; MCPS, Mexico City Prospective Study; UKB, UK Biobank; VAF, variant allele frequency.

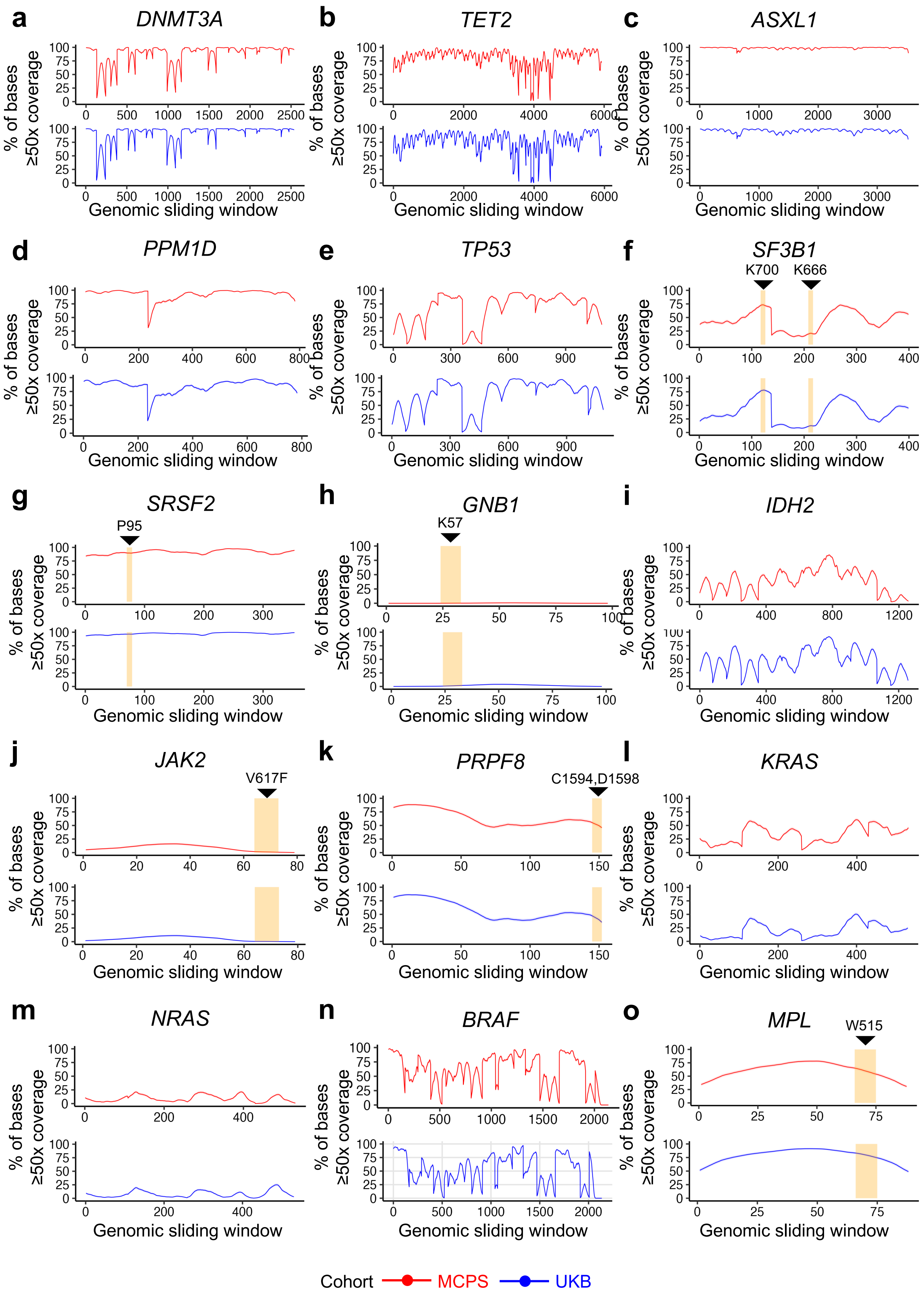

a DNMT3A
b TET2
c ASXL1
d PPM1D
e TP53
f SF3B1
K700
K666
g SRSF2
P95
h GNB1
K57
i IDH2
j JAK2
V617F
k PRPF8
C1594,D1598
l KRAS
m NRAS
n BRAF
o MPL
W515
% of bases ≥50x coverage
Genomic sliding window
Cohort
MCPS
UKB

**Supplementary Fig. 2 | Comparison of coverage distribution across the coding regions of clonal haematopoiesis (CH) genes between Mexico City Prospective Study (MCPS) and UK Biobank (UKB). (a-o)** One-thousand samples were randomly selected from MCPS and UKB each, for analysis here. For each gene, the genomic coordinates were binned into 10bp sliding window, and for each window, the percentage of bases with ≥50x coverage were computed. For the following genes, only specific regions of the isoforms were assessed for coverage: *ASXL1* (exons 12 and 13), *PPM1D* (exons 5 and 6), *SF3B1* (exons 14 and 15 in which the K666 and K700 hotspot variants are located, respectively), *SRSF2* (exon 1 in which the P95 hotspot variant is located), *GNB1* (exon 5 in which the K57 hotspot variant is located), *JAK2* (exon 14 in which the V617F hotspot variant is located), *PRPF8* (exon 31 in which the C1594 and D1598 hotspot variants are located), and *MPL* (exon 10 in which the W515 hotspot variant is located). The genomic sliding windows in which the hotspots are located are highlighted in peach colour. For all other genes, the entire coding region of the isoform was assessed for coverage. CH, clonal haematopoisis; MCPS, Mexico City Prospective Study; UKB, UK Biobank.

a

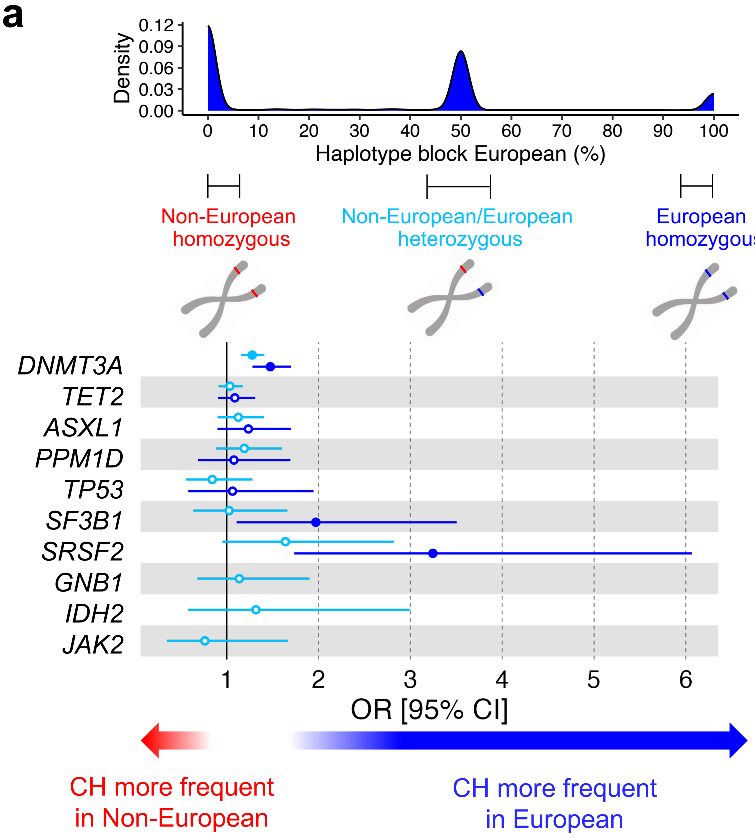


| | Additive OR [95% CI] | Additive *P* value |
|---|---|---|
| *DNMT3A* | 1.23 [1.15,1.31] | $3.79 \times 10^{-9}$ |
| *TET2* | 1.04 [0.96,1.13] | 0.35 |
| *ASXL1* | 1.12 [0.96,1.29] | 0.14 |
| *PPM1D* | 1.08 [0.89,1.32] | 0.44 |
| *TP53* | 0.97 [0.73,1.28] | 0.82 |
| *SF3B1* | 1.33 [0.99,1.79] | 0.060 |
| *SRSF2* | 1.79 [1.30,2.46] | $3.24 \times 10^{-4}$ |
| *GNB1* | 1.05 [0.74,1.49] | 0.79 |
| *IDH2* | 1.15 [0.67,1.99] | 0.61 |
| *JAK2* | 0.64 [0.34,1.18] | 0.15 |

b

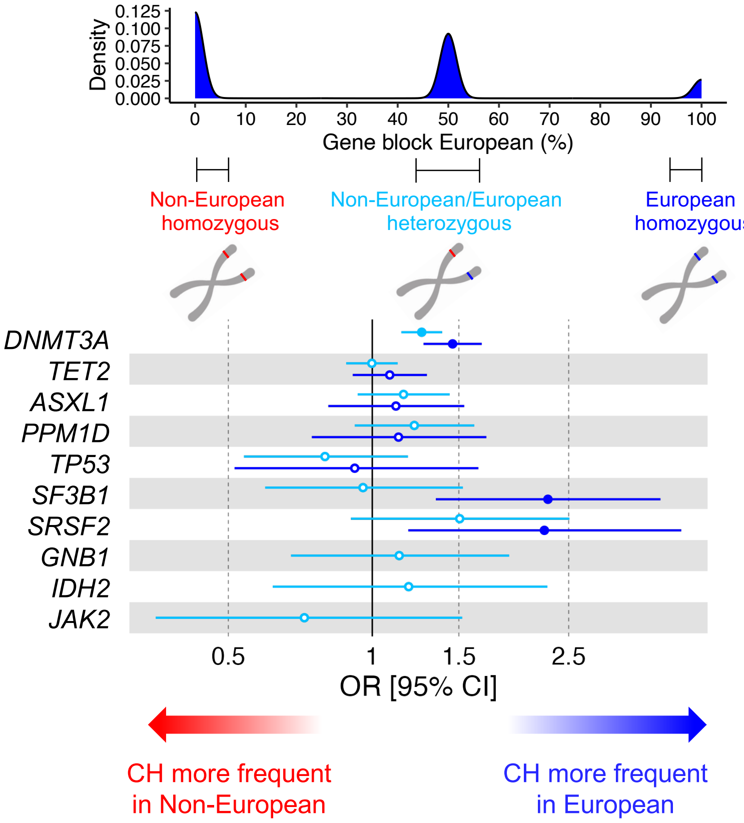


| | Additive OR [95% CI] | Additive *P* value |
|---|---|---|
| *DNMT3A* | 1.22 [1.15,1.30] | $4.99 \times 10^{-10}$ |
| *TET2* | 1.03 [0.95,1.12] | 0.48 |
| *ASXL1* | 1.08 [0.94,1.25] | 0.27 |
| *PPM1D* | 1.10 [0.92,1.32] | 0.30 |
| *TP53* | 0.90 [0.69,1.18] | 0.46 |
| *SF3B1* | 1.42 [1.07,1.87] | 0.014 |
| *SRSF2* | 1.50 [1.10,2.04] | 0.011 |
| *GNB1* | 1.14 [0.81,1.60] | 0.46 |
| *IDH2* | 1.00 [0.64,1.56] | 0.99 |
| *JAK2* | 0.69 [0.39,1.20] | 0.19 |

**Supplementary Fig. 3 | Prevalence of gene-specific clonal haematopoiesis (CH) in Mexico City Prospective Study (MCPS) participants with European (homozygous or heterozygous) versus non-European (homozygous American or African) haplotype block or gene block in which the corresponding CH driver gene is located**. **(a,b)** Odds ratios and unadjusted two-sided *P* values were derived from a logistic regression model with gene-specific CH as outcome, and RFMix-inferred ancestry at haplotype block **(a)** or gene block level **(b)** as predictor, adjusted for age, sex, and smoking status. In total, 134,255 individuals with RFMix-inferred ancestry and smoking status available were included for analysis here. Measures of centre represent the odds ratios, and the error bars represent the lower and upper bound of the 95% confidence interval of the odds ratios. Full circles represent significant associations ($P < 0.05$) while hollow circles represent non-significant associations ($P \geq 0.05$). CH, clonal haematopoiesis; CI, confidence interval; MCPS, Mexico City Prospective Study; OR, odds ratio.

**a**

| | | rs10131341 (*TCL1A* upstream) | |
|---|---|---|---|
| | | A | C |
| rs187319135 (*TCL1B* upstream) | C | 78.29% | 20.72% |
| | T | 0.98% | 0.01% |

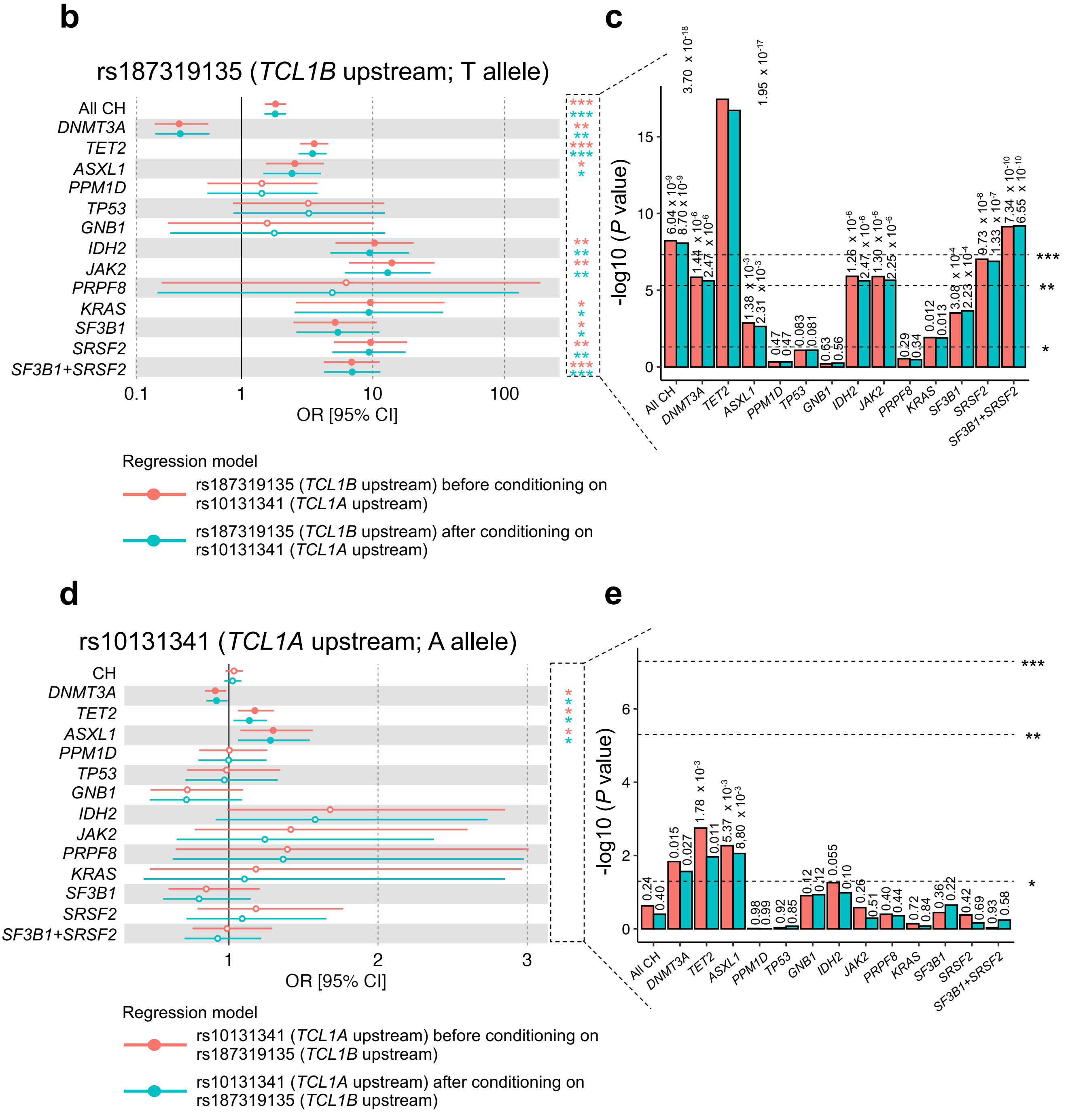

**Supplementary Fig. 4 | Linkage and conditional analysis of rs187319135 (*TCL1B* upstream) and rs10131341 (*TCL1A* upstream) variants in Mexico City Prospective Study (MCPS). a,** Phasing of rs187319135 and rs10131341 using PLINK2. These variants are in high linkage disequilibrium (LD) with each other (D' = 0.95, $r^2$ = 0.0024). **b,c**, Risk conferred by rs187319135 (T allele) to overall clonal haematopoiesis (CH) and gene-specific CH before versus after conditioning on rs10131341. Odds ratios and unadjusted two-sided *P* values were derived from Firth logistic regression implemented by REGENIE software, adjusted for age, sex, and first ten genetic principal components. In total, 136,401 MCPS participants with complete co-variate data available were included for analysis here. Measures of centre represent the odds ratios, and the error bars represent the lower and upper bound of the 95% confidence interval of the odds ratios. Full circles represent significant associations ($P < 0.05$) while hollow circles represent non-significant associations ($P \geq 0.05$). **d,e**, Risk conferred by rs10131341 (A allele) to overall CH and gene-specific CH before versus after conditioning on rs187319135. Odds ratios and unadjusted two-sided *P* values were derived from Firth logistic regression implemented by REGENIE software, adjusted for age, sex, and first ten genetic principal components. In total, 136,401 MCPS participants with complete co-variate data available were included for analysis here. Measures of centre represent the odds ratios, and the error bars represent the lower and upper bound of the 95% confidence interval of the odds ratios. Full circles represent significant associations ($P < 0.05$) while hollow circles represent non-significant associations ($P \geq 0.05$). *P* value *** < $5 \times 10^{-8}$ (genome-wide significant), ** < $5 \times 10^{-6}$ (suggestive), * < 0.05 (nominal). 95% CI, 95% confidence interval; CH, clonal haematopoiesis; LD, linkage disequilibrium; MCPS, Mexico City Prospective Study; OR, odds ratio.

**a**

| | | rs2887399 (*TCL1A* promoter) G | rs2887399 (*TCL1A* promoter) T |
|---|---|---|---|
| rs187319135 (*TCL1B* upstream) | C | 84.91% | 14.10% |
| | T | 9.87% | 0.004% |

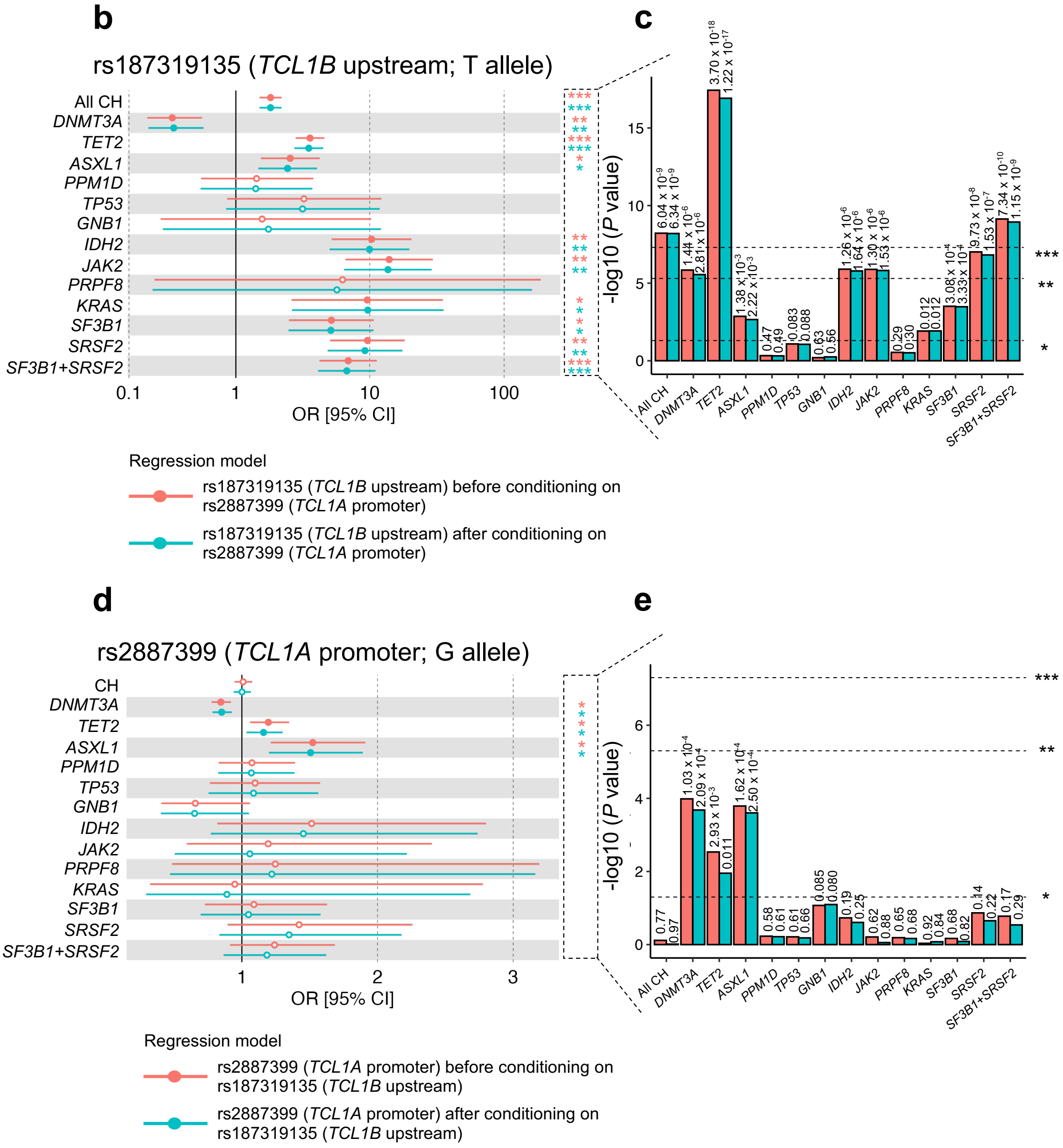

**Supplementary Fig. 5 | Linkage and conditional analysis of rs187319135 (*TCL1B* upstream) and rs2887399 (*TCL1A* upstream) variants in Mexico City Prospective Study (MCPS). a,** Phasing of rs187319135 and rs2887399 using PLINK2. These variants are in high linkage disequilibrium (LD) with each other (D' = 0.97, $r^2$ = 0.0016). **b,c**, Risk conferred by rs187319135 (T allele) to overall clonal haematopoiesis (CH) and gene-specific CH before versus after conditioning on rs2887399. Odds ratios and unadjusted two-sided *P* values were derived from Firth logistic regression implemented by REGENIE software, adjusted for age, sex, and first ten genetic principal components. In total, 136,401 MCPS participants with complete co-variate data available were included for analysis here. Measures of centre represent the odds ratios, and the error bars represent the lower and upper bound of the 95% confidence interval of the odds ratios. Full circles represent significant associations ($P < 0.05$) while hollow circles represent non-significant associations ($P \geq 0.05$). **d,e**, Risk conferred by rs2887399 (G allele) to overall CH and gene-specific CH before versus after conditioning on rs187319135. Odds ratios and unadjusted two-sided *P* values were derived from Firth logistic regression implemented by REGENIE software, adjusted for age, sex, and first ten genetic principal components. In total, 136,401 MCPS participants with complete co-variate data available were included for analysis here. Measures of centre represent the odds ratios, and the error bars represent the lower and upper bound of the 95% confidence interval of the odds ratios. Full circles represent significant associations ($P < 0.05$) while hollow circles represent non-significant associations ($P \geq 0.05$). *P* value *** < $5 \times 10^{-8}$ (genome-wide significant), ** < $5 \times 10^{-6}$ (suggestive), * < 0.05 (nominal). 95% CI, 95% confidence interval; CH, clonal haematopoiesis; LD, linkage disequilibrium; MCPS, Mexico City Prospective Study; OR, odds ratio.

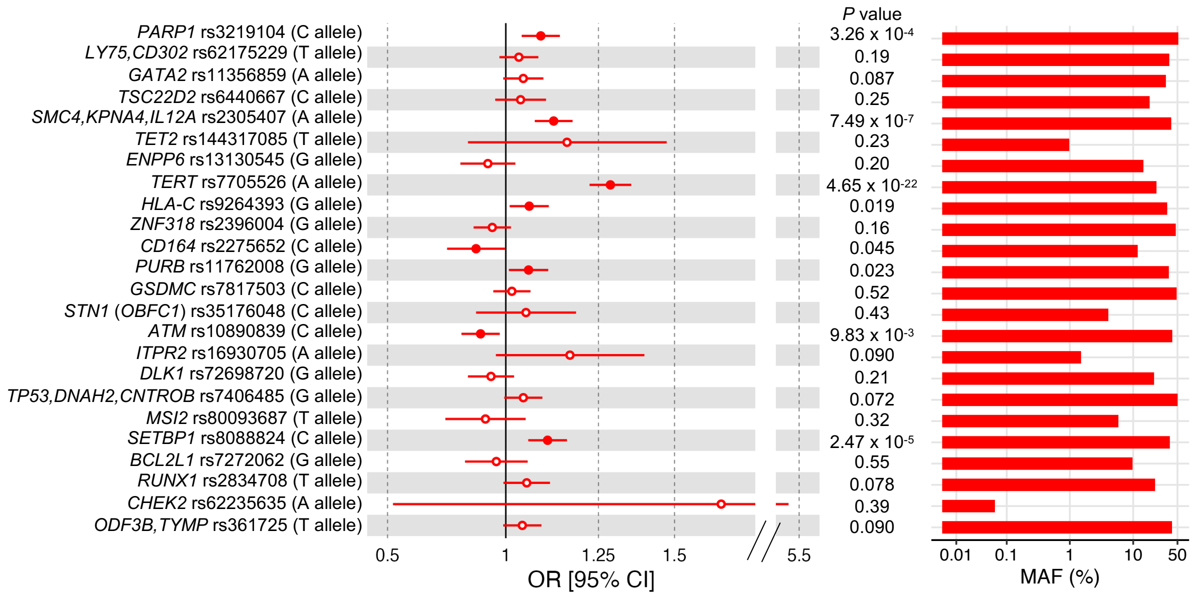

P value
PARP1 rs3219104 (C allele) 3.26 x 10-4
LY75,CD302 rs62175229 (T allele) 0.19
GATA2 rs11356859 (A allele) 0.087
TSC22D2 rs6440667 (C allele) 0.25
SMC4,KPNA4,IL12A rs2305407 (A allele) 7.49 x 10-7
TET2 rs144317085 (T allele) 0.23
ENPP6 rs13130545 (G allele) 0.20
TERT rs7705526 (A allele) 4.65 x 10-22
HLA-C rs9264393 (G allele) 0.019
ZNF318 rs2396004 (G allele) 0.16
CD164 rs2275652 (C allele) 0.045
PURB rs11762008 (G allele) 0.023
GSDMC rs7817503 (C allele) 0.52
STN1 (OBFC1) rs35176048 (C allele) 0.43
ATM rs10890839 (C allele) 9.83 x 10-3
ITPR2 rs16930705 (A allele) 0.090
DLK1 rs72698720 (G allele) 0.21
TP53,DNAH2,CNTROB rs7406485 (G allele) 0.072
MSI2 rs80093687 (T allele) 0.32
SETBP1 rs8088824 (C allele) 2.47 x 10-5
BCL2L1 rs7272062 (G allele) 0.55
RUNX1 rs2834708 (T allele) 0.078
CHEK2 rs62235635 (A allele) 0.39
ODF3B,TYMP rs361725 (T allele) 0.090
0.5 1 1.25 1.5 5.5
OR [95% CI]
0.01 0.1 1 10 50
MAF (%)

**Supplementary Fig. 6 | Summary statistics of Mexico City Prospective Study (MCPS) genome-wide association study (GWAS) of overall clonal haematopoiesis (CH) for common risk variants previously identified from European populations.** Risk estimates conferred by reported risk variants and minor allele frequency (MAF) for the respective risk variants estimated from our study indicated. Odds ratios and unadjusted two-sided *P* values were derived from Firth logistic regression implemented by REGENIE software, adjusted for age, sex, and first ten genetic principal components. In total, 136,401 MCPS participants with complete co-variate data available were included for analysis here. Measures of centre represent the odds ratios, and the error bars represent the lower and upper bound of the 95% confidence interval of the odds ratios. Full circles represent significant associations ($P < 0.05$) while hollow circles represent non-significant associations ($P \geq 0.05$). 95% CI, 95% confidence interval; CH, clonal haematopoiesis; GWAS, genome-wide association study; MAF, minor allele frequency; MCPS, Mexico City Prospective Study; OR, odds ratio.

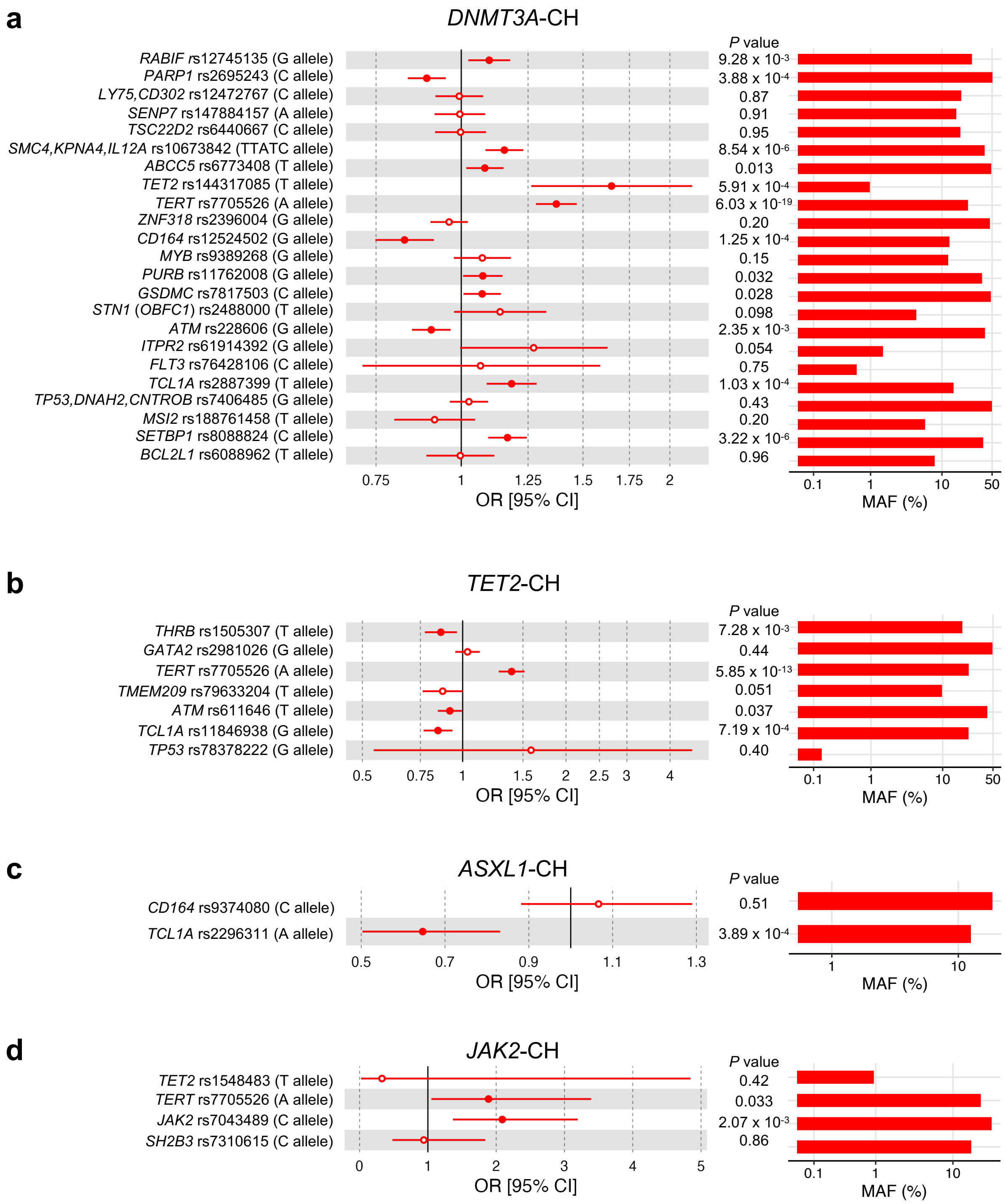
a
DNMT3A-CH
P value
RABIF rs12745135 (G allele)
PARP1 rs2695243 (C allele)
LY75,CD302 rs12472767 (C allele)
SENP7 rs147884157 (A allele)
TSC22D2 rs6440667 (C allele)
SMC4,KPNA4,IL12A rs10673842 (TTATC allele)
ABCC5 rs6773408 (T allele)
TET2 rs144317085 (T allele)
TERT rs7705526 (A allele)
ZNF318 rs2396004 (G allele)
CD164 rs12524502 (G allele)
MYB rs9389268 (G allele)
PURB rs11762008 (G allele)
GSDMC rs7817503 (C allele)
STN1 (OBFC1) rs2488000 (T allele)
ATM rs228606 (G allele)
ITPR2 rs61914392 (G allele)
FLT3 rs76428106 (C allele)
TCL1A rs2887399 (T allele)
TP53,DNAH2,CNTROB rs7406485 (G allele)
MSI2 rs188761458 (T allele)
SETBP1 rs8088824 (C allele)
BCL2L1 rs6088962 (T allele)
9.28 x 10-3
3.88 x 10-4
0.87
0.91
0.95
8.54 x 10-6
0.013
5.91 x 10-4
6.03 x 10-19
0.20
1.25 x 10-4
0.15
0.032
0.028
0.098
2.35 x 10-3
0.054
0.75
1.03 x 10-4
0.43
0.20
3.22 x 10-6
0.96
0.75 1 1.25 1.5 1.75 2
OR [95% CI]
0.1 1 10 50
MAF (%)
b
TET2-CH
P value
THRB rs1505307 (T allele)
GATA2 rs2981026 (G allele)
TERT rs7705526 (A allele)
TMEM209 rs79633204 (T allele)
ATM rs611646 (T allele)
TCL1A rs11846938 (G allele)
TP53 rs78378222 (G allele)
7.28 x 10-3
0.44
5.85 x 10-13
0.051
0.037
7.19 x 10-4
0.40
0.5 0.75 1 1.5 2 2.5 3 4
OR [95% CI]
0.1 1 10 50
MAF (%)
c
ASXL1-CH
P value
CD164 rs9374080 (C allele)
TCL1A rs2296311 (A allele)
0.51
3.89 x 10-4
0.5 0.7 0.9 1.1 1.3
OR [95% CI]
1 10
MAF (%)
d
JAK2-CH
P value
TET2 rs1548483 (T allele)
TERT rs7705526 (A allele)
JAK2 rs7043489 (C allele)
SH2B3 rs7310615 (C allele)
0.42
0.033
2.07 x 10-3
0.86
0 1 2 3 4 5
OR [95% CI]
0.1 1 10
MAF (%)

**Supplementary Fig. 7 | Summary statistics of Mexico City Prospective Study (MCPS) genome-wide association study (GWAS) of gene-specific overall clonal haematopoiesis (CH) for common risk variants previously identified from European populations. a-d**, Risk estimates conferred by reported risk variants for *DNMT3A*- (**a**), *TET2*- (**b**), *ASXL1*- (**c**), and *JAK2*- (**d**) CH. The MAF for the respective risk variants estimated from our study also indicated. Odds ratios and unadjusted two-sided *P* values were derived from Firth logistic regression implemented by REGENIE software, adjusted for age, sex, and first ten genetic principal components. In total, 136,401 MCPS participants with complete co-variate data available were included for analysis here. Measures of centre represent the odds ratios, and the error bars represent the lower and upper bound of the 95% confidence interval of the odds ratios. Full circles represent significant associations ($P < 0.05$) while hollow circles represent non-significant associations ($P \geq 0.05$). 95% CI, 95% confidence interval; CH, clonal haematopoiesis; GWAS, genome-wide association study; MCPS, Mexico City Prospective Study; MAF, minor allele frequency; OR, odds ratio.

**a**

rs774615666 (*TCL1B* promoter)

| rs187319135 (*TCL1B* upstream) | C/C | C/T | T/T |
|---|---|---|---|
| C/C | 132,414 | 105 | 0 |
| C/T | 1,458 | 664 | 0 |
| T/T | 5 | 2 | 3 |

**b**

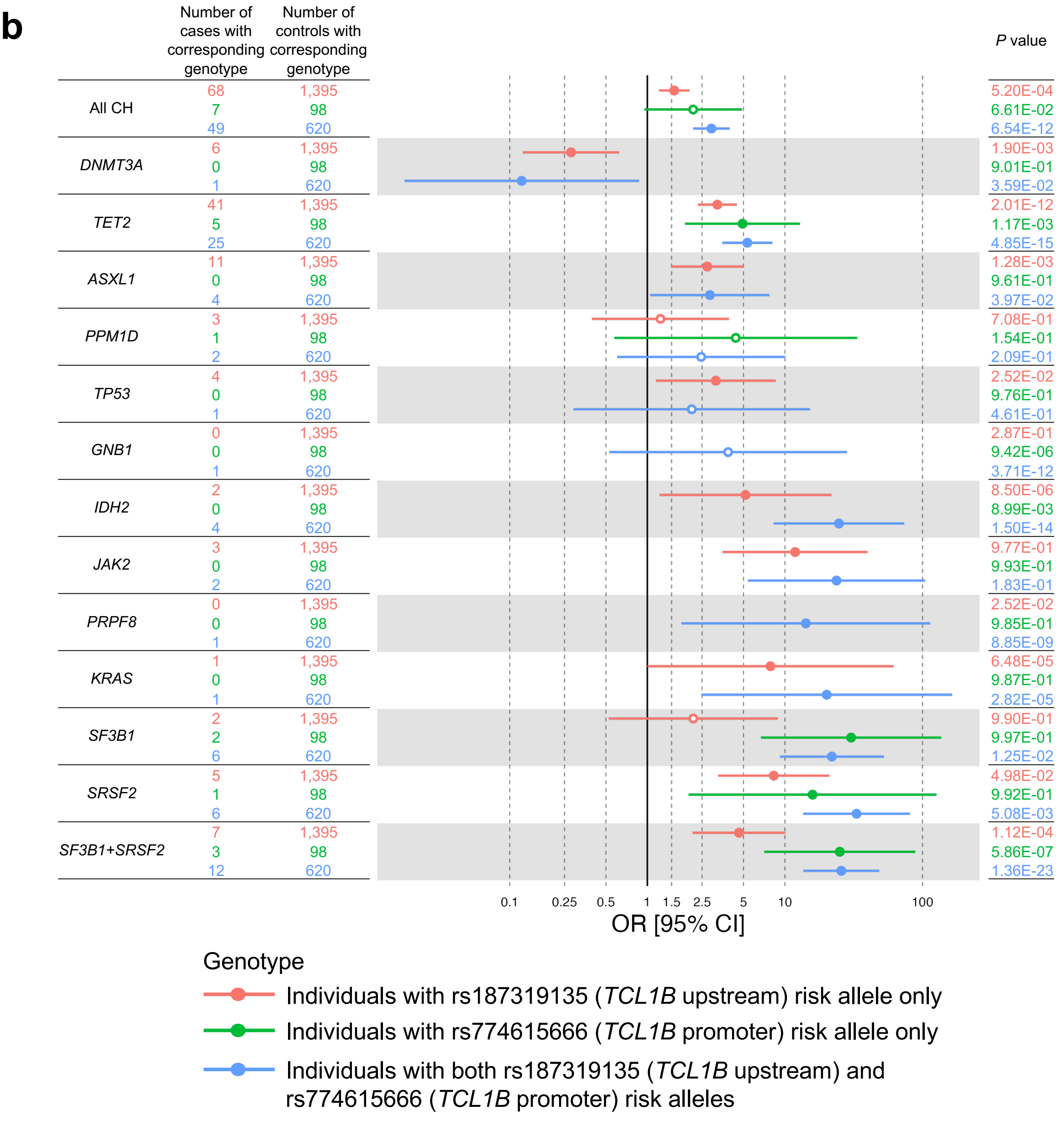

**Supplementary Fig. 8 | Overall clonal haematopoiesis (CH) and gene-specific CH risk conferred by genotypes based on rs187319135 (*TCL1B* upstream) and rs774615666 (*TCL1B* promoter) in Mexico City Prospective Study (MCPS). a,** Cross tabulation of the rs187319135 and rs774615666 genotypes. **b**, Overall CH and gene-specific CH risk estimates in individuals with rs187319135 risk (T) allele only, individuals with rs774615666 risk (T) allele only, and individuals with both rs187319135 and rs774615666 risk alleles relative to individuals with no risk alleles for both rs187319135 and rs774615666. Odds ratios and unadjusted two-sided *P* values were derived from logistic regression model with all CH or gene-specific CH as outcome, rs187319135/rs774615666 genotype as predictor, adjusted for age, sex, and first ten principal components. In total, 134,651 participants with SNP array-based hard-called rs187319135 genotype, whole-exome sequencing (WES)-called rs774615666 genotype available, and complete co-variate data available were included for analysis here. Measures of centre represent the odds ratios, and the error bars represent the lower and upper bound of the 95% confidence interval of the odds ratios. Full circles represent significant associations ($P < 0.05$) while hollow circles represent non-significant associations ($P \geq 0.05$). 95% CI, 95% confidence interval; CH, clonal haematopoiesis; MCPS, Mexico City Prospective Study; OR, odds ratio; WES, whole-exome sequencing.

## Supplementary Methods

**Admixture mapping**

Admixture mapping was performed by assessing the association between all genomic windows with overall CH and gene-specific CH. Genomic windows with *P* value < 1.25 x $10^{-6}$ (0.05/39,861 genomic windows) were considered as genome-wide significant. Age, sex, smoking status, and global European ancestry were included as covariates and logistic regression was performed using the *glm* function as implemented by the *stats* package in R (v4.2.2).

## Supplemental Notes

**Supplemental Note 1: Association between haplotype- and gene-level ancestry with overall CH and gene-specific CH among MCPS participants.**

We observed increased risk of overall CH and gene-specific CH (*DNMT3A*, *ASXL1*, and *SRSF2*) in MCPS participants with high proportion of European genome (Fig. 2d). We therefore sought to assess if individuals with high proportion of European genome at the haplotype block and gene locus are also at higher risk of the corresponding CH driver gene. Interestingly, European ancestry of the haplotype block and gene locus in which the CH gene resides positively correlated with frequency of *DNMT3A*-, *SF3B1*-, and *SRSF2*-CH (Supplementary Fig. 3a and b). Notably, individuals with homozygous European ancestry were associated with higher frequency of CH compared to individuals with heterozygous European ancestry (for example, homozygous European ancestry at *DNMT3A* haplotype block: OR = 1.48 [1.28,1.70], $P = 7.79 \times 10^{-8}$; heterozygous European ancestry at *DNMT3A* haplotype block: OR = 1.28 [1.16,1.41], $P = 1.09 \times 10^{-6}$). However, this was largely explained by correlation between local (haplotype- and gene-level) and global (genome-wide) genetic ancestry (Supplementary Table 10). For example, association between *DNMT3A* haplotype ancestry and *DNMT3A*-CH was attenuated, but nevertheless remained significant, after including global ancestry in our model (beta = 0.21 [0.14,0.27], $P = 3.79 \times 10^{-10}$, and 0.09 [0.02-0.17], $P = 0.016$, before and after including global ancestry as covariate, respectively). Therefore, while haplotype- and gene-level ancestry may appear to contribute to CH risk, global ancestry plays a larger role in determining CH risk. Correspondingly, admixture mapping did not identify additional genomic intervals associated with CH at genome-wide significance (defined as $P < 1.25 \times 10^{-6}$).

**Supplementary Note 2: Delineating independence of CH risk associated with *TCL1B* variant (rs187319135) relative to *TCL1A* variants (rs10131341 and rs2887399) among MCPS participants.**

We identified the minor allele of a novel variant upstream of *TCL1B* (rs187319135) to be associated with increased risk to non-DNMT3A CH but decreased risk to *DNMT3A*-CH (Fig. 4b). This is reminiscent of the major allele of the previously reported *TCL1A* upstream (rs10131341) and promoter (rs2887399) risk variants that are associated with increased risk to non-DNMT3A CH, specifically *TET2*- and *ASXL1*-CH, but decreased risk to *DNMT3A*-CH[1-3]. The minor allele of rs187319135 is often co-inherited with the major allele of rs10131341 and rs2887399 (D' = 0.95 and 0.97, respectively; Supplementary Fig. 4a and 5a). However, it is noteworthy that the overall allele correlation between rs187319135 with rs10131341 and rs2887399 was low ($r^2$ = 0.0024 and $r^2$ = 0.0016, respectively). This is due to *TCL1B* risk variants being rarer relative to *TCL1A* risk variants in MCPS. Correspondingly, rs187319135 remained associated with overall CH, and *TET2*- and *SF3B1*+*SRSF2*-CH at genome-wide significance threshold even after conditioning on rs10131341 and rs2887399 in MCPS (Supplementary Fig. 4b-c and 5b-c). We also replicated the association between rs10131341 and rs2887399 with *DNMT3A*-, *TET2*-, and *ASXL1*-CH at nominal significance threshold ($P$ < 0.05), and the association remained significant after conditioning on rs187319135 (Supplementary Fig. 4d-e and 5d-e). Taken together, our observations suggest independence of CH risk attributed to *TCL1B* and *TCL1A* risk variants among MCPS participants.

**Supplementary Note 3: Delineating independence of CH risk associated with *TCL1B* upstream variant (rs187319135) relative to *TCL1B* promoter variant (rs774615666) among MCPS participants.**

In our CH GWAS, we identified the minor allele of a *TCL1B* upstream variant (rs187319135) to associated with increased risk to non-DNMT3A CH but decreased risk to DNMT3A-CH (Fig. 4b). In our CH exome-wide association study (ExWAS), we further identified the minor allele of a *TCL1B* promoter variant (rs774615666) to be similarly associated with increased risk to non-DNMT3A CH but decreased risk to *DNMT3A*-CH (Fig. 4d). To delineate the overall CH and gene-specific CH risks conferred by these variants, we stratified individuals into those with rs187319135 risk allele only, rs774615666 risk allele only, and both rs187319135 and rs774615666 risk alleles and compared them with individuals with no risk alleles for both rs187319135 and rs774615666 (Supplementary Fig. 8a). We observed individuals carrying only the rs187319135 risk allele to be at higher risk of overall CH and *TET2*-, *ASXL1*-, *TP53*-, *IDH2*-, *JAK2*-, *KRAS*-, *SRSF2*-, and *SF3B1*+*SRSF2*-CH, but decreased risk to *DNMT3A*-CH ($P$ < 0.05, Supplementary Fig. 8b). We further observed individuals carrying only the rs774615666 risk allele to be at increased risk of *TET2*-, *SF3B1*-, *SRSF2*-, and *SRSF2*-*SF3B1*-CH ($P$ < 0.05). The residual associations observed after conditioning the *TCL1B* upstream and promoter variants on each other suggest partial independence of these risk variants.

**Supplementary References**


1. Kar, S.P. *et al.* Genome-wide analyses of 200,453 individuals yield new insights into the causes and consequences of clonal hematopoiesis. *Nat Genet* **54**, 1155-1166 (2022).
2. Kessler, M.D. *et al.* Common and rare variant associations with clonal haematopoiesis phenotypes. *Nature* **612**, 301-309 (2022).
3. Weinstock, J.S. *et al.* Aberrant activation of TCL1A promotes stem cell expansion in clonal haematopoiesis. *Nature* **616**, 755-763 (2023).
