# Supplementary material for: Comparative analysis of the Mexico City Prospective Study and the UK Biobank identifies ancestry-specific effects on clonal hematopoiesis
Source: Nat Genet. 2025 Feb 13;57(3):572–82. doi: 10.1038/s41588-025-02085-6 (PMC11906367; doi:10.1038/s41588-025-02085-6)
Supplement: Supplementary file 2 — Reporting Summary [file 41588_2025_2085_MOESM2_ESM.pdf]

nature portfolio

Corresponding author(s): Dr Jonathan Mitchell
Prof. George Vassiliou

Last updated by author(s): Dec 3, 2024

Reporting Summary

Nature Portfolio wishes to improve the reproducibility of the work that we publish. This form provides structure for consistency and transparency in reporting. For further information on Nature Portfolio policies, see our Editorial Policies and the Editorial Policy Checklist.

Statistics

For all statistical analyses, confirm that the following items are present in the figure legend, table legend, main text, or Methods section.

n/a	Confirmed	
☐	☒	The exact sample size (n) for each experimental group/condition, given as a discrete number and unit of measurement
☐	☒	A statement on whether measurements were taken from distinct samples or whether the same sample was measured repeatedly
☐	☒	The statistical test(s) used AND whether they are one- or two-sided<br>*Only common tests should be described solely by name; describe more complex techniques in the Methods section.*
☐	☒	A description of all covariates tested
☐	☒	A description of any assumptions or corrections, such as tests of normality and adjustment for multiple comparisons
☐	☒	A full description of the statistical parameters including central tendency (e.g. means) or other basic estimates (e.g. regression coefficient) AND variation (e.g. standard deviation) or associated estimates of uncertainty (e.g. confidence intervals)
☐	☒	For null hypothesis testing, the test statistic (e.g. F, t, r) with confidence intervals, effect sizes, degrees of freedom and P value noted<br>*Give P values as exact values whenever suitable.*
☒	☐	For Bayesian analysis, information on the choice of priors and Markov chain Monte Carlo settings
☒	☐	For hierarchical and complex designs, identification of the appropriate level for tests and full reporting of outcomes
☐	☒	Estimates of effect sizes (e.g. Cohen's d, Pearson's r), indicating how they were calculated

Our web collection on statistics for biologists contains articles on many of the points above.

Software and code

Policy information about availability of computer code

Data collection

No software was used for data collection.

Data analysis

All analyses were performed using publicly available software and web-based applications as indicated in the Methods section.
1. Conversion of sequencing data in BCL format to FASTQ format: bcl2fastq v2.19.0
2. Sequencing read alignment to the GRCh38 genome reference and germline variant detection: Illumina DRAGEN Bio-IT Platform Germline Pipeline v3.0.7
3. Somatic variant calling: GATK MuTect2 v.2.2.0
4. DNA sample contamination: VerifyBAMID
5. Kinship: KING v2.2.3
6. Ancestry probability: peddy v0.4.2
7. Genome- and exome-wide association studies (GWAS and ExWAS, respectively): REGENIE v3.5
8. Whole-genome inferred telomere length: TelSeq v.0.0.2
9. Polygenic risk scoring, linkage disequilibrium analysis: PLINK2.0
10. Local ancestry inference: RFMix2.0
11. Logistic regression: glm function as implemented in stats package in R v4.2.20
12. Cross-ancestry GWAS and ExWAS meta-analysis: METAL (released 2011-03-25)
13. Gene-level collapsing analysis: Cochran-Mantel-Haenszel (CMH) test using the mantelhaen.test function as implemented by the stats package in R v4.2.2

For manuscripts utilizing custom algorithms or software that are central to the research but not yet described in published literature, software must be made available to editors and reviewers. We strongly encourage code deposition in a community repository (e.g. GitHub). See the Nature Portfolio guidelines for submitting code & software for further information.

Data

Policy information about availability of data

All manuscripts must include a data availability statement. This statement should provide the following information, where applicable:

- Accession codes, unique identifiers, or web links for publicly available datasets
- A description of any restrictions on data availability
- For clinical datasets or third party data, please ensure that the statement adheres to our policy

Full summary statistics for GWAS are available on NHGRI-EBI GWAS Catalog while the full summary statistics for ExWAS and gene-collapsing analysis are available on Zenodo. The GWAS Catalog accession numbers and Zendo links are indicated in Supplementary Table 23. Individual-level UK Biobank data may be requested via applicable to the UK Biobank. Individual-level MCPS data may be requested via Data and Sample Access Policy available on the study's Oxford-hosted webpage (http://www.ctsu.ox.ac.uk/research/mcps).

Research involving human participants, their data, or biological material

Policy information about studies with human participants or human data. See also policy information about sex, gender (identity/presentation), and sexual orientation and race, ethnicity and racism.

Reporting on sex and gender	Sex concordance was determined by comparing clinically-reported sex against chromosome X:Y consensus coding sequence coverage ratio. Only sex, but not gender, was reported by this study.
Reporting on race, ethnicity, or other socially relevant groupings	Ancestry of study participants were determined using the peddy software. Europeans individuals from UK Biobank were defined with peddy-inferred European probability of at least 95%. Similarly, Admixed American individuals from Mexico City Prospective Study (MCPS) were defined with peddy-inferred Admixed American probability of at least 95%. Neither race nor ethnicity was reported by this study.
Population characteristics	MCPS is a prospective cohort of more than 150,000 adults with genetic (whole-genome sequencing, whole-exome sequencing, SNP array) and phenotypic, and metabolomic data available. Details have been described in Ziyatdinov et al. (Nature, 2023) and Tapia-Conyer et al. (International Journal of Epidemiology, 2006). UKB is a prospective cohort of approximately 500,000 adults with genetic (whole-genome sequencing, whole-exome sequencing, SNP array) and phenotypic, proteomic, and matobolomic data available. Details for UKB have been described in Szustakowski et al. (Nature Genetics, 2021) and Bycroft et al. (Nature, 2018).<br><br>The median age of MCPS and UKB participants are 58 and 51 years old, respectively. In MCPS, female and male constitute 67% and 33% of participants, respectively. In UKB, female and male constitute 54% and 46% of participants, respectively.
Recruitment	MCPS participants were aged at least 35 years, and recruited between 1998 and 2004 from the contiguous urban districts of Coyoacán and Iztapalapa in Mexico City. UKB participants were aged between 40 to 70 years, and recruited since 2007.
Ethics oversight	The MCPS study was approved by the Mexican Ministry of Health, the Mexican National Council for Science and Technology, and the University of Oxford, and the UKB study has approval from the North-West Multi-centre Research Ethics Committee (11/NW/0382).

Note that full information on the approval of the study protocol must also be provided in the manuscript.

Field-specific reporting

Please select the one below that is the best fit for your research. If you are not sure, read the appropriate sections before making your selection.

☒ Life sciences ☐ Behavioural & social sciences ☐ Ecological, evolutionary & environmental sciences

For a reference copy of the document with all sections, see nature.com/documents/nr-reporting-summary-flat.pdf

Life sciences study design

All studies must disclose on these points even when the disclosure is negative.

Sample size	Initial 141,046 individuals from MCPS and 469,809 individuals from UKB were identified on the basis of whole-exome sequencing data available.
Data exclusions	In both MCPS and UKB, samples were selected on the basis of (1) contamination <4% computed by VerifyBAMID software, (2) gender concordant between clinically reported and chromosome X:Y consensus coding sequence (CCDS) coverage ratios, (3) ≥94.15% of CCDS r22 bases covered with ≥10x coverage, (4) within 4SDs of mean genetic principal components 1-4 as computed by the peddy software, and (5) SNP array QC (genotype missingness ≤10%). Samples from MCPS were additionally selected based on within 2 standard deviations (SDs) of the mean read-depth distribution, no pairs with kinship >0.45, and probability ≥0.95 of Admixed American ancestry. Samples from UKB were additionally selected based on no pairs with kinship >0.1769 and probability ≥0.95 of European ancestry. Kinship and and ancestry were inferred using the KING and peddy softwares, respectively. peddy Admixed American and European ancestry probabilities were computed with the 1000 Genomes Admixed American and European reference panel, respectively. For UKB, individuals with prior diagnosis of

	haematological malignancies were excluded. Post-QC, 136,401 individuals from MCPS and 416,118 individuals from UKB were included in our study.
Replication	Non-applicable. This is a non-experimental, descriptive population-based study.
Randomization	Non-applicable. This is a non-experimental, descriptive population-based study.
Blinding	Non-applicable. This is a non-experimental, descriptive population-based study.

Reporting for specific materials, systems and methods

We require information from authors about some types of materials, experimental systems and methods used in many studies. Here, indicate whether each material, system or method listed is relevant to your study. If you are not sure if a list item applies to your research, read the appropriate section before selecting a response.

Materials & experimental systems

n/a	Involved in the study
☒	☐ Antibodies
☒	☐ Eukaryotic cell lines
☒	☐ Palaeontology and archaeology
☒	☐ Animals and other organisms
☒	☐ Clinical data
☒	☐ Dual use research of concern
☒	☐ Plants

Methods

n/a	Involved in the study
☒	☐ ChIP-seq
☒	☐ Flow cytometry
☒	☐ MRI-based neuroimaging

Plants

Seed stocks	*Report on the source of all seed stocks or other plant material used. If applicable, state the seed stock centre and catalogue number. If plant specimens were collected from the field, describe the collection location, date and sampling procedures.*
Novel plant genotypes	*Describe the methods by which all novel plant genotypes were produced. This includes those generated by transgenic approaches, gene editing, chemical/radiation-based mutagenesis and hybridization. For transgenic lines, describe the transformation method, the number of independent lines analyzed and the generation upon which experiments were performed. For gene-edited lines, describe the editor used, the endogenous sequence targeted for editing, the targeting guide RNA sequence (if applicable) and how the editor was applied.*
Authentication	*Describe any authentication procedures for each seed stock used or novel genotype generated. Describe any experiments used to assess the effect of a mutation and, where applicable, how potential secondary effects (e.g. second site T-DNA insertions, mosiacism, off-target gene editing) were examined.*
